# Differential *In Vitro* Effects of Intravenous versus Oral Formulations of Silibinin on the HCV Life Cycle and Inflammation

**DOI:** 10.1371/journal.pone.0016464

**Published:** 2011-01-28

**Authors:** Jessica Wagoner, Chihiro Morishima, Tyler N. Graf, Nicholas H. Oberlies, Elodie Teissier, Eve-Isabelle Pécheur, John E. Tavis, Stephen J. Polyak

**Affiliations:** 1 Department of Laboratory Medicine, University of Washington, Seattle, Washington, United States of America; 2 Department of Microbiology, University of Washington, Seattle, Washington, United States of America; 3 Department of Global Health, University of Washington, Seattle, Washington, United States of America; 4 Department of Chemistry and Biochemistry, University of North Carolina at Greensboro, Greensboro, North Carolina, United States of America; 5 Institut de Biologie et Chimie des Protéines, UMR CNRS 5086, Université Lyon 1, IFR128 Lyon Biosciences Gerland, Lyon, France; 6 CNRS-Universite Claude Bernard, Lyon, France; 7 Department of Molecular Microbiology and Immunology, Saint Louis University School of Medicine, St. Louis, Missouri, United States of America; McMaster University, Canada

## Abstract

Silymarin prevents liver disease in many experimental rodent models, and is the most popular botanical medicine consumed by patients with hepatitis C. Silibinin is a major component of silymarin, consisting of the flavonolignans silybin A and silybin B, which are insoluble in aqueous solution. A chemically modified and soluble version of silibinin, SIL, has been shown to potently reduce hepatitis C virus (HCV) RNA levels in vivo when administered intravenously. Silymarin and silibinin inhibit HCV infection in cell culture by targeting multiple steps in the virus lifecycle. We tested the hepatoprotective profiles of SIL and silibinin in assays that measure antiviral and anti-inflammatory functions. Both mixtures inhibited fusion of HCV pseudoparticles (HCVpp) with fluorescent liposomes in a dose-dependent fashion. SIL inhibited 5 clinical genotype 1b isolates of NS5B RNA dependent RNA polymerase (RdRp) activity better than silibinin, with IC50 values of 40–85 µM. The enhanced activity of SIL may have been in part due to inhibition of NS5B binding to RNA templates. However, inhibition of the RdRps by both mixtures plateaued at 43–73%, suggesting that the products are poor overall inhibitors of RdRp. Silibinin did not inhibit HCV replication in subgenomic genotype 1b or 2a replicon cell lines, but it did inhibit JFH-1 infection. In contrast, SIL inhibited 1b but not 2a subgenomic replicons and also inhibited JFH-1 infection. Both mixtures inhibited production of progeny virus particles. Silibinin but not SIL inhibited NF-κB- and IFN-B-dependent transcription in Huh7 cells. However, both mixtures inhibited T cell proliferation to similar degrees. These data underscore the differences and similarities between the intravenous and oral formulations of silibinin, which could influence the clinical effects of this mixture on patients with chronic liver diseases.

## Introduction

Globally, HCV infects an estimated 150 million people, and causes an estimated 376,000 deaths per year due to complications of end stage liver disease [1]. In the United States, about 1.8% of the general population (∼4 million persons) is infected. Pegylated interferon (IFN) plus ribavirin therapy is now the standard of care [2], [3], [4]. However, 50% of treated patients still do not clear viremia when treated with peg-IFN plus ribavirin. Moreover, IFN therapy is costly, has significant side effects, and many patients are ineligible for therapy. Thus, many patients seek complementary and alternative medicine (CAM)-based strategies to improve their health.

Silymarin, which consists primarily of seven flavonolignans [5], is an extract of the seeds of milk thistle Silybum marianum, and is the most common botanical consumed by patients with chronic hepatitis C [6]. Many effects of silymarin have been described *in vitro* and in animal models, all of which likely contribute to its hepatoprotective effects. These include anti-oxidant, anti-inflammatory, anti-proliferative, anti-fibrotic, anti-viral and immunomodulatory effects [7]. The description of these effects in modern journals, in addition to descriptions in ancient medical texts and oral history have likely influenced the millions of people throughout the world who consume silymarin, despite the fact that clinical trials have shown variable clinical efficacy [8], [9], [10], and mechanisms of action in chronic hepatitis C have only begun to be characterized.

We have shown that silymarin and a major component of the extract, silibinin, which is composed of silybin A and silybin B [5], inhibits HCV replication, HCV-induced oxidative stress, NF-κB dependent transcription, and T cell proliferation and inflammatory cytokine production[11], [12], [13]. Silymarin also inhibits NS5B RNA dependent RNA polymerase (RdRp) activity in *in vitro* assays using purified recombinant polymerase. However, polymerase inhibition is variable [14] [11], [15].

Silibinin is insoluble in aqueous solution. Moreover, silymarin and silibinin are typically administered orally, which limits the efficacy of the natural product because of its poor absorption and short half-life in the body. Indeed, plasma levels of silymarin-derived flavonolignans peak within 1–2 hours of ingestion, and are gone from circulation 4–6 hours later [16]. In Germany, an intravenous formulation of silibinin, Legalon-SIL (SIL), is licensed for toxic mushroom poisoning [17]. SIL is a water-soluble version of silibinin containing two succinate moieties covalently attached to both silybin A and silybin B.

Recently SIL has been shown to potently reduce HCV RNA levels in vivo when administered intravenously [18], [19]. By contrast, silymarin and silibinin do not appear to reduce HCV RNA levels in patients when ingested orally [20], [21], although they both inhibit HCV infection in cell culture [11], [12], [15]. Since silibinin and SIL are chemically different compounds, we hypothesized that they exhibit differential effects. We therefore compared the hepatoprotective profiles of the natural product silibinin with SIL in assays that measure antiviral and anti-inflammatory functions.

## Materials and Methods

### Subjects

Healthy subjects gave written informed consent to donate blood through a University of Washington (UW) Institutional Review Board-approved protocol; peripheral blood mononuclear cells (PBMC) from these samples were used to generate the in vitro results reported here.

### Cells

Human hepatoma Huh7 and Huh7.5.1 cells were grown in Huh7 medium as described [12]. BB7 and SGR7 cells are Huh7 cell lines that contain subgenomic genotype 1b and 2a (JFH1) replicons [11], [22]. JFH-1 viral stock preparation, cell infection and titration was performed as described [12]. PBMC were freshly isolated using standard Ficoll-Hypaque centrifugation within 24 hours of venipuncture and immediately applied to the assays described below.

### PBMC Proliferation Assay

PBMC were stimulated for 1 day at 37°C 5%CO_2_ with plate-bound anti-CD3 (UCHT1, 10 µg/mL, BD Biosciences, San Jose, CA) in RPMI 1640 medium supplemented with 10% human serum (Gemini Bio-Products, Woodland, CA). Cellular proliferation was detected by ^3^H-thymidine incorporation into replicating DNA was measured by adding 1 µCi to each replicate well of 10^5^ PBMC for 24 hours before quantitative analysis using a Topcount® Liquid Scintillation Counter (Perkin-Elmer, Waltham, MA). Data are presented as mean counts per minute (cpm) incorporated per condition tested. All experiments utilized three to four replicates per condition.

### Silibinin and SIL

**Figure 1:**
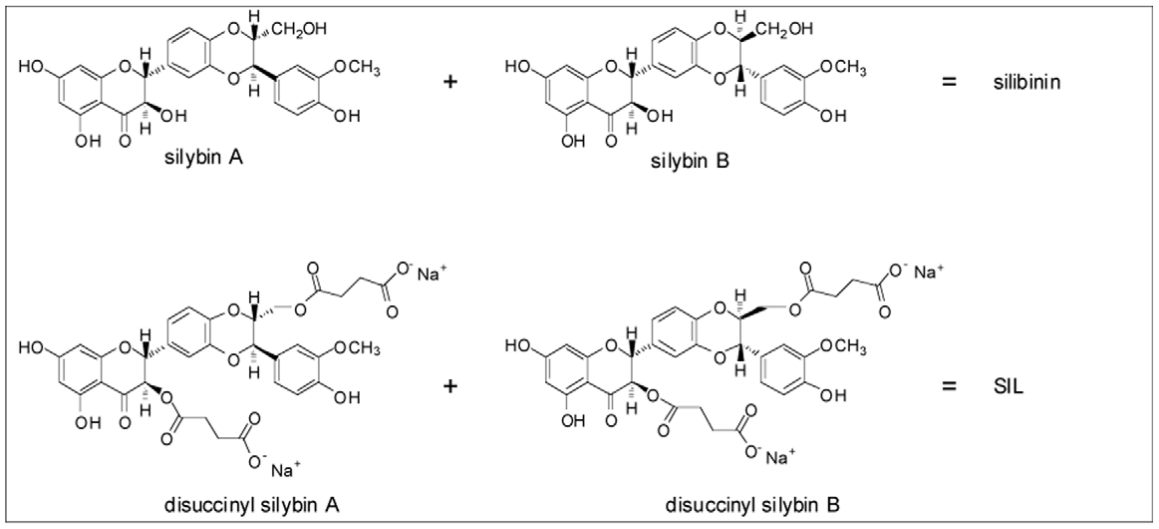
Chemical structures of SIL and silibinin. See text for details.

Silibinin was purified from silymarin as described [23]. SIL was kindly provided by Drs. Ulrich Mengs and Joe Villeaux (Rottapharm/Madaus). Silibinin was solubilized in dimethylsulfoxide (DMSO) for hepatocyte or methanol for PBMC experiments. SIL was solubilized in PBS. Silibinin was an equimolar mixture of silybin A and silybin B, and SIL was an equimolar mixture of disodium disuccinyl silybin A and disodium disuccinyl silybin B (Figure 1).

### Western Blot Analysis

Western blots were performed as described [12]. Actin and NS5A were detected using commercial antiserum (Santa Cruz Biotechnology, Santa Cruz, CA; BioDesign/Meridian Life Science, Saco, ME). NS5A in JFH-1 infected cells was detected with serum from patients infected with HCV genotype 2a.

### HCV RNA Quantitation

HCV RNA was quantitated by real time RT-PCR, as previously described [24]. Briefly, RNA was extracted from cells following 72 hours of culture with SIL, silibinin, or PBS or DMSO solvent controls using a commercial kit (Qiagen). Ten nanograms of RNA were added to wells of a 384 well plate and the RT-PCR reaction performed on an ABI HT7900 real time RT-PCR machine. For each run, dilutions of BB7 or JFH-1 plasmid DNA (precisely quantitated using the RiboGreen DNA quantitation kit (Invitrogen)) ranging from 0–10^7^ copies per well were run in triplicate to generate a standard curve, which served as a reference to calculate HCV RNA copy number based on the cycle threshold (Ct).

### Infectious Virus Production

Supernatants were harvested from cell cultures treated with SIL, silibinin, or relevant solvent controls (PBS or DMSO), and filtered through 10,000 molecular weight cutoff filters (UFC501096 Amicon Ultra-0.5, Ultracel-10 Membrane) to remove carry over of SIL and silibinin. Filtered supernatants were serially diluted in Huh7 media and used to infect naïve Huh7.5.1 cells. Seventy-two hours later, cells were fixed and the HCV core protein was detected by immunofluorescence, using previously described methods [15].

### Membrane Fusion Assays

Lipid mixing between HCVpp and PC:chol:R_18_ liposomes was monitored by fluorescent spectroscopy, as the dequenching of R_18_ [25]. In brief, R_18_-labeled liposomes were added to a well of a 37°C-thermostable quartz microplate, containing 20 mL of HCVpp in PBS pH 7.4 or PBS pH 7.4/0.5% DMSO, and incubated 2 min. Lipid mixing was initiated by acidification to pH 5.0 with diluted HCl, and recorded on a Tecan Infinite® M1000 spectrofluorimeter over a 15-min time period, at λ_exc_ = 560 nm and λ_em_ = 590 nm. Maximal R_18_ dequenching was measured after the addition of 0.1% Triton X-100 (final concentration) to the well. The same procedure was used to follow HCVpp fusion in the presence of silibinin in DMSO or SIL in PBS. After a 1-min incubation of HCVpp with liposomes, silibinin (at 10.4, 20.7, 82.8 µM) or SIL (at 6.9, 13.8 or 55.2 µM) final concentration was added and incubated for 1 min, and fusion initiated by acidification.

### HCV NS5B Polymerase Assays

NS5BΔC21 RNA polymerases C-terminally fused to a hexa-histidine tag were expressed and purified as described [26]. RNA polymerase activity was measured as poly-G synthesis from primed poly-C templates as described (6). All reaction components except [α^32^P]GTP and silibinin or SIL were preincubated for 30 minutes at 30°C, then the reaction was started by adding [α^32^P]GTP plus silibinin or SIL and was incubated for 1.5 hours at 30°C. Reaction products were collected on nitrocellulose filters, the filters were washed five times, air-dried, and subjected to liquid scintillation counting to quantify the retained ^32^P-labeled RNA. All measurements were done in triplicate and the IC50 values were calculated with GraphPad Prism.

### RNA Binding Assays

RNA binding by recombinant NS5B RNA polymerase was determined using a filter-binding assay as previously described [26]. Briefly, the polymerase was incubated with radiolabeled RNAs for 30 minutes on ice and the mixture was then passed through Hybond-ECL nylon-backed nitrocellulose (GE Healthcare), and Hybond-N (GE Healthcare) membranes using a slot-blot apparatus. The membranes were dried and retained radioactivity was quantified with a Storm phosphorimager (GE Healthcare).

### Cytotoxicity Determinations

The toxicity of SIL on BB7 and Huh7.5.1 cells was determined by measuring ATP levels using the ATPlite system (Perkin Elmer, Boston, MA), as described [12].

PBMC viability was assessed by measuring cellular ATP levels using the ATPlite kit (Perkin Elmer) and also by labeling with Live/Dead Fixable Dead Cell Stain Kit (Violet viability dye, Invitrogen/Molecular Probes, Carlsbad, CA) after culture/stimulation for 24 hours and analysis using a Becton Dickinson FACS Calibur (Puget Sound Blood Center, Seattle, WA) and FlowJo software for Macintosh (version 6.3.3, Treestar, Inc., Ashland, OR). ATPlite results are reported in relative light units (RLU).

### Reporter Gene Assays

Endotoxin free plasmid DNA was purified (Plasmid Midi Kit + Endofree buffers, QIAGEN, Valencia, CA), and was introduced into cells with Lipofectamine 2000 as described [27]. 100 ng of the pRDII-luciferase gene was transfected into cells in quadruplicate. Eighteen hours later, cells were preincubated with SIL or silibinin for 30 minutes before rhTNF-α (10 ng/ml; Sigma Aldrich, St. Louis, MO) was added. Four hours later, luciferase activity was measured on cell lysates using the Britelite Assay System (Perkin Elmer). In separate experiments 50 ng/well of an IFN-B promoter-luciferase plasmid was co-transfected with 25 ng of IRF3-5D, a constitutively active mutant of IRF-3[28], and 24 hours later, cells were treated with silibinin or SIL for an additional 24 hours before luciferase activity was measured. Results are reported in relative light units (RLU).

## Results

The chemical composition of silibinin and SIL are illustrated in Figure 1. The preparations were compared on an equimolar basis in all experiments.

### Cytotoxicity Profile of SIL

**Figure 2:**
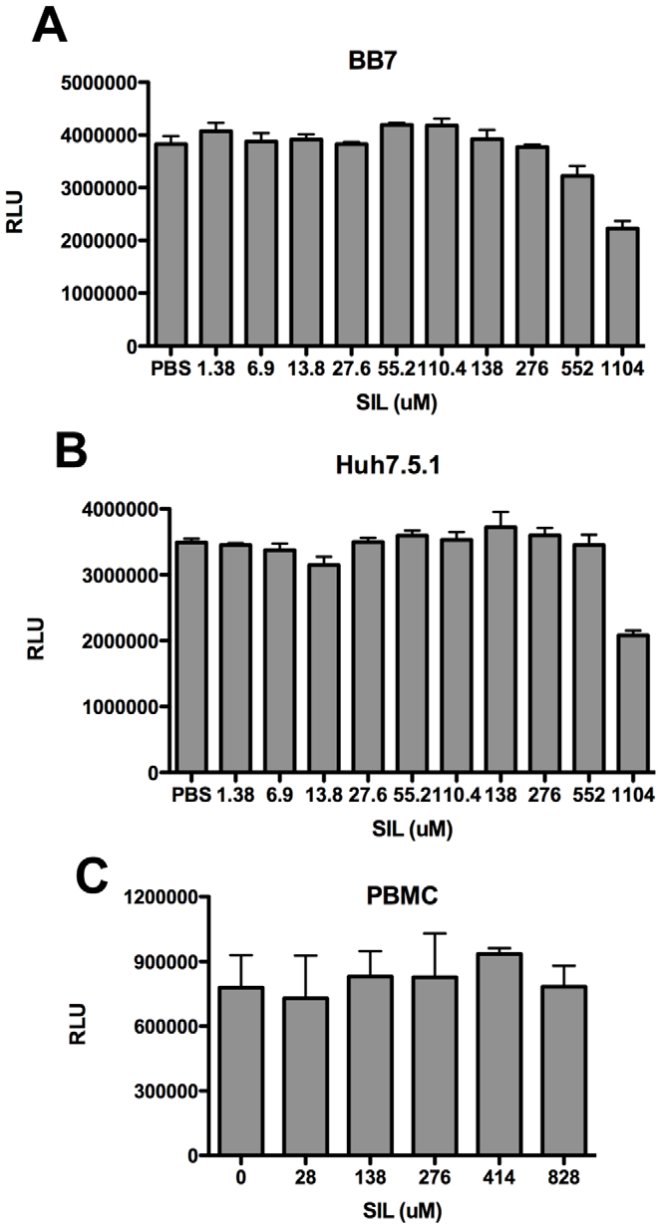
Cytotoxicity profile of SIL on BB7 subgenomic replicon cells (A), Huh7.5.1 cells (B), and PBMC (C). Cells were plated in quadruplicate in 96 well plates and the indicated concentrations of SIL in PBS were added. Cells were incubated for 72 hours (for BB7 and Huh7.5.1 cells) and 24 hours for PBMCs before ATP levels were measured as described in the Materials and Methods. Fluorescence is reported as relative light units (RLU).

We first determined the cytotoxicity profile of SIL, by measuring cellular ATP levels, which is a sensitive marker for cytotoxicity, using a fluorescence-based assay (please see Materials and Methods). SIL was well tolerated by BB7 subgenomic replicon (Figure 2A) and Huh7.5.1 cells (Figure 2B) over 500 µM. Similarly, PBMC viability was maintained at high concentrations of SIL, with only a 25% decrease in viability (measured as a reduction in relative light units (RLU)) at a dose of 828 µM (Figure 2C). This is strikingly different than silibinin, which is toxic to BB7, Huh7.5.1, and PBMC above 80 µM [15]. Cytotoxicity against PBMC was also evaluated by uptake of a viability dye and no significant toxicity to SIL was noted through 828 µM (data not shown).

### Antiviral Effects of SIL and Silibinin: Effects on Fusion

**Figure 3:**
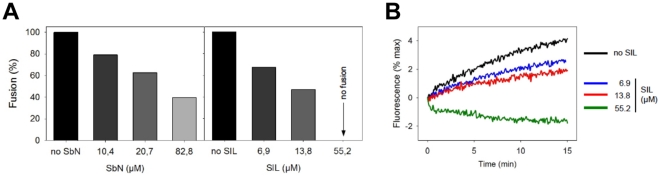
Silibinin and SIL inhibit HCVpp-mediated fusion. Membrane fusion between HCVpp and R_18_-labeled liposomes was followed by fluorescence spectroscopy with excitation and emission at 560 and 590 nm, respectively. Fluorescent liposomes (12.5 µM final lipid concentration) were added to 20 µl of HCVpp in PBS pH 7.4 at 37°C, in the absence or presence of 5, 10 or 40 µg/ml of indicated compound, which corresponds to 6.9, 13.8 or 55 µM of SIL and 10.4, 20.7, and 82.8 µM of silibinin. After a 2 min-equilibration, lipid mixing was initiated by decreasing the pH to 5.0 with diluted HCl, and R_18_ dequenching was recorded. Maximal fluorescence was obtained after addition of 0.1% final Triton X-100 to the cuvette. A, values of the last min of fusion (final extent of fusion) were used to calculate the percentage of fusion in the presence of the drug, relative to 100% fusion in the absence of drug. Results are expressed from the mean of 2 separate experiments. Compounds were added at 5 (black), 10 (dark grey) or 40 µg/ml (light grey). B, fusion kinetics of HCVpp with liposomes, in the absence (black) or presence of three concentrations of SIL: blue, 5 µg/ml; red, 10 µg/ml and green, 40 µg/ml.

During HCV entry into liver cells, the viral envelope fuses with endosomal membranes to release the viral genome into cells [29]. We have recently shown that silymarin and other synthetic antivirals block the fusion process [11], [30]. We therefore tested silibinin and SIL in a lipid mixing experiment. In this assay, HCVpp are mixed with fluorescently-labeled liposomes, in the presence or absence of silibinin or SIL. Mixing of viral envelope lipids with liposomes, defined as the fluorescence dequenching of the probe incorporated in the liposome membrane, is then measured by spectrofluorimetry. Both mixtures inhibited fusion, observed as a decrease in fluorescence recovery, in a dose-dependent manner, with SIL being more potent (Figure 3A). Indeed, SIL completely inhibited fusion at 55 µM, with an IC50 of approximately 6 µM (Figure 3B). Note that DMSO, the solvent into which silibinin is dissolved, did not affect fusion (data not shown). The data suggest that silibinin and SIL can inhibit HCV entry at the fusion stage.

### Antiviral Effects of SIL and Silibinin: Effects on HCV Polymerase

**Figure 4:**
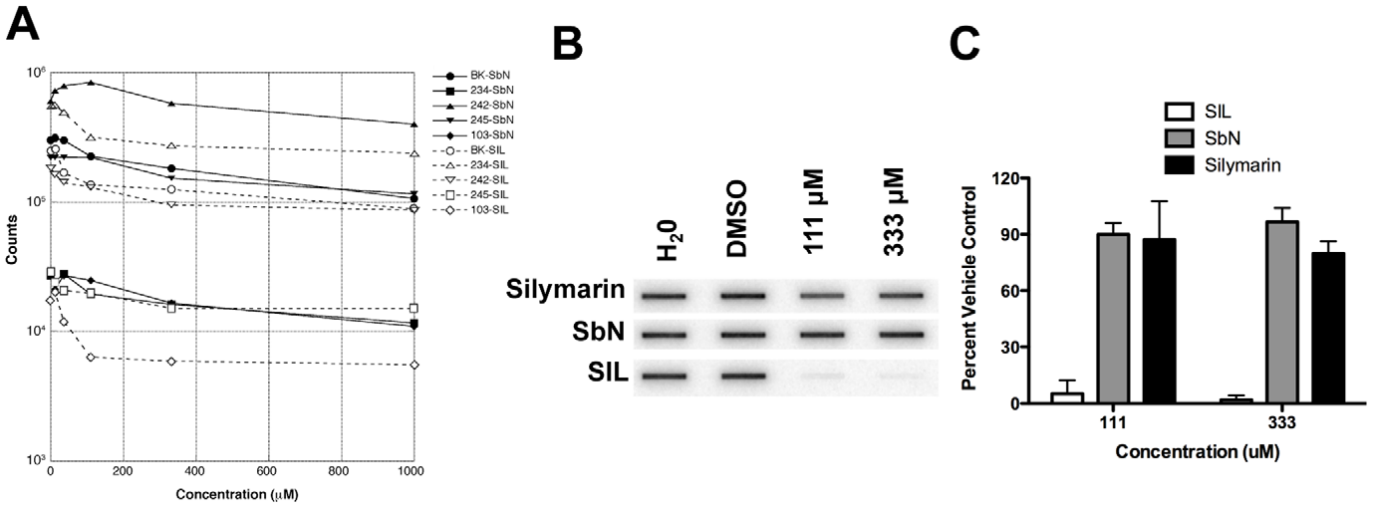
Effect of silibinin and SIL on HCV RdRp Activity and Binding to RNA. A, Effects of silibinin and SIL on RNA synthesis by the HCV RdRp. Purified HCV subtype 1b RdRps from the reference isolate BK and 4 patient derived-isolates were preincubated with poly-C and G10, then [α 32P]GTP and varying concentrations of silibinin or SIL were added and the reactions were incubated to permit RNA synthesis. The 32P-labeled RNA was collected on nitrocellulose filters and retained radioactivity was measured by scintillation counting. Solid black lines, filled symbols, and SbN indicate silibinin-containing reactions; open symbols, dashed lines, and SIL indicate SIL containing reactions. Concentrations are in micromolar. B, Example RNA binding assay measuring the effect of silymarin, SbN, and SIL on RNA binding by the HCV RNA polymerase. Purified RNA polymerase from the reference strain BK was allowed to bind to a 32P-labeled RNA in the presence of varying concentrations of the drugs, the mixture was passed through a nitrocellulose filter to collect protein:RNA complexes, the filter was washed, and retained RNA was detected by phosphorimage analysis. C, Summary of 4 independent repeats of the RNA binding assays. All data are normalized to values obtained with the DMSO vehicle control, and error bars represent the standard deviation of the measurements.

Figure 4A and Table 1 compare the ability of silibinin and SIL to inhibit the standard BK genotype 1b NS5B RNA polymerase and 4 patient-derived 1b isolates with a wide range of basal RNA polymease activities [31] by SIL and silibinin. As described in the Materials and Methods, the assay measures the ability of purified NS5B proteins to incorporate 32P labeled GTP into a synthetic RNA template. The inhibitory activity of silibinin against all 5 RdRps was weak and was similar to that of silymarin [11], with IC50s ranging from 169 to >1000 µM. SIL inhibited RNA synthesis by the RdRps more efficiently, as indicated by lower IC50 values (40–85 µM).

**Table 1:** Inhibition of NS5B RNA Dependent RNA Polymerase (RdRp) Activity by SIL and silibinin (SbN).

RdRp	Drug	Max inhibition (%)	IC50 (µM)
BK	SIL	58.8	39.9
	SbN	67.3	210.0
234	SIL	43.1	43.9
	SbN	57.0	169.0
242	SIL	57.3	64.3
	SbN	45.7	∼1000
245	SIL	49.5	84.9
	SbN	58.8	359.0
103	SIL	72.8	40.2
	SbN	69.3	354.0

Polymerase assays were conducted as described in the Materials and Methods.

To further investigate the difference in activity between SIL and silibinin, the effect of these mixtures on the ability of purified NS5B RNA polymerase to bind 32P-labeled RNA templates was measured in a filter-binding assay. Figures 4B and 4C demonstrates that SIL inhibited RNA binding by the polymerase at lower concentration than they inhibited RNA polymerization, with approximately 90% inhibition by 111 µM. In contrast, neither silymarin nor SbN inhibited RNA binding strongly at these concentrations. The data indicate that SIL was able to prevent NS5B binding to RNA templates *in vitro*. However, as shown in Table 1, the net inhibition of all polymerases when the drugs were added after the RNA polymerase was allowed to bind to the primer template was very limited because the inhibition plateaued at 43–73% even at high drug concentrations. Together, these data indicate that SIL may inhibit HCV replication in part by blocking binding of the RNA polymerase to its template, but that direct inhibition of the RNA polymerase activity is unlikely to be a major contributor to its antiviral effect.

### Antiviral Effects of SIL and Silibinin: Effects on HCV RNA and Protein Expression in Replicon Cells and JFH-1 Infected Cells

**Figure 5:**
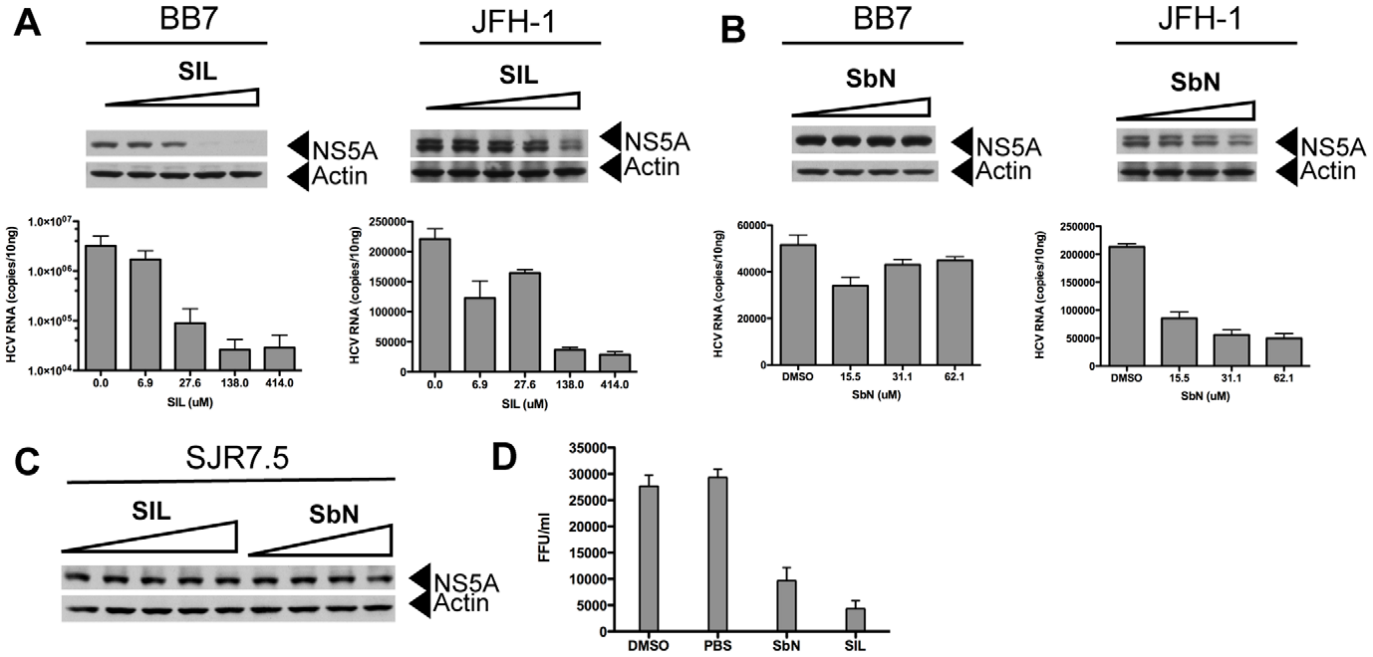
Antiviral Activity of SIL and silibinin. A, effect of SIL on HCV replication in genotype 1b subgenomic replicon cells (BB7) and JFH-1 infection of Huh7.5.1 cells. The top panels show HCV NS5A protein expression detected by western blot, while the lower graph depicts HCV RNA levels determined by real time RT-PCR. Cells were treated with 0, 6.9, 27.6, 138, and 414 µM of SIL for 72 hours before protein and RNA isolations. B, effect of silibinin on HCV replication in genotype 1b subgenomic replicon cells (BB7) and JFH-1 infection of Huh7.5.1 cells. Cells were treated with DMSO, 15.5, 31.1, and 62.1 µM of silibinin for 72 hours before protein and RNA isolations. C, effects of SIL and silibinin on HCV replication in subgenomic JFH-1 replicon cells. Cells were treated with 0, 6.9, 27.6, 138, and 414 µM of SIL or DMSO, 20.7, 41.4, and 82.8 µM of silibinin for 72 hours before proteins were extracted and NS5A detected by western blot. D, effect of SIL and silibinin on progeny virus production. Huh7.5.1 cells were treated with 20 µg/ml silibinin, 300 µg/ml SIL or DMSO and PBS controls immediately after 5 hours of adsorption with JFH-1 at an m.o.i. of 0.05. Culture supernatants were harvested 72 hours later and carry over silibinin or SIL was removed by concentration through 10,000 molecular filters. Supernatants were diluted 1∶100 in Huh7 media and used to infect naïve Huh7.5.1 cells in triplicate and immunofluorescent detection of HCV core protein was performed. Foci were counted manually and used to calculate infectious virus yields expressed as focus forming units per milliliter (FFU/ml). Error bars represent standard deviations of triplicate cultures.

Figure 5 shows the antiviral profiles of SIL (Figure 5A) and silibinin (Figure 5B) against BB7, a subgenomic 1b replicon cell line, and against JFH-1 infection of Huh7.5.1 cells. SIL inhibited HCV RNA and protein expression in BB7 replicon cells above 25 µM. SIL inhibited JFH-1 RNA and protein expression at higher concentrations of 138 and 414 µM. In contrast, silibinin did not significantly inhibit HCV RNA and protein expression in BB7 replicon cells, but inhibited JFH-1 RNA and protein expression above 15 µM (Figure 5B and [11], [15]). Furthermore, neither SIL nor silibinin inhibited viral protein expression in subgenomic JFH-1 replicon cell lines (Figure 5C). Thus, the antiviral profile of silibinin is identical to silymarin [11], which inhibits HCV infection but does not inhibit HCV replication in non-infectious replicon cell lines. SIL inhibits genotype 1b non-infectious replicons and JFH-1 infection, with stronger antiviral activity against the genotype 1b replicon. Silibinin and SIL also inhibited infectious progeny virus production, measured as a decrease in infectivity titers (focus-forming units per milliliter) in supernatants from infected cells (Figure 5D).

### Effects of SIL and Silibinin on Inflammatory and Antiviral Signaling

**Figure 6:**
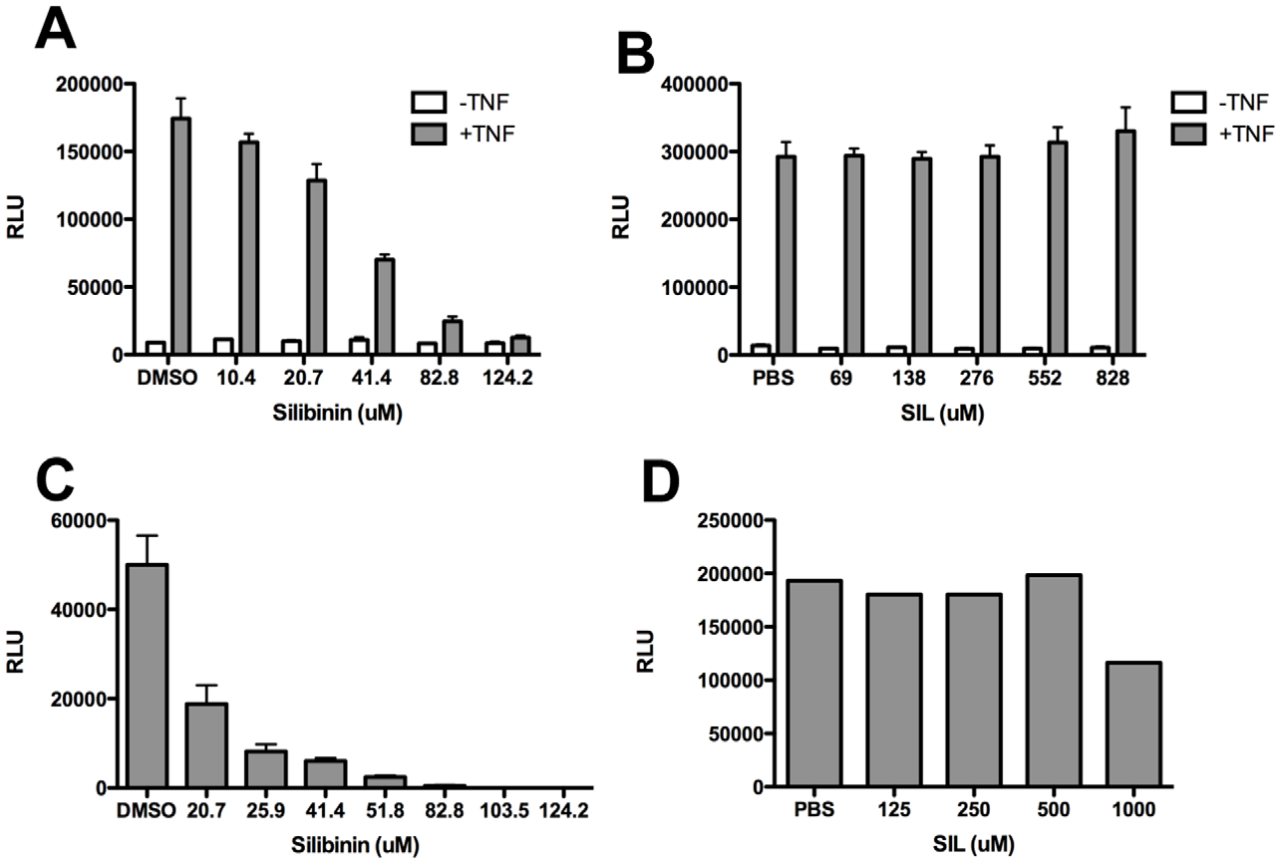
Silibinin but not SIL inhibits innate inflammatory and antiviral signaling. A, B, effect of silibinin and SIL on NF-κB dependent transcription. Huh7 cells were transfected with an NF-κB responsive reporter plasmid (pRDII-luc) and twenty-four hours later, cells were pretreated with the indicated doses of silibinin (A) or SIL (B). Cells were then treated with 10 ng/ml TNF-α and luciferase activity measured by Britelite assay 3.5 hours later. C, D, effect of silibinin and SIL on IRF-3 driven transcription from the IFN-B promoter. Huh7.5.1 cells were co-transfected with a luciferase reporter plasmid under control of the IFN-B promoter and IRF-35D, a constitutively active mutant of IRF-3 [28]. Twenty-four hours later, cells were pretreated with the indicated doses of silibinin (C) or SIL (D). Luciferase activity measured by Britelite assay 24 hours later. Fluorescence is reported as relative light units (RLU). Error bars represent standard deviation from triplicate cultures.

Silymarin suppresses inflammatory cytokine and chemokine induction via blockade of NF-κB [11], [12], [13], [15]. We therefore compared the ability of SIL and silibinin to block NF-κB using a luciferase reporter gene under control of the NF-κB promoter. Figures 6A and 6B demonstrate that silibinin but not SIL blocked TNF-α induced NF-κB transcription. Next, to examine the effect of SIL and silibinin on innate antiviral signaling, we measure the effect of the mixtures on IRF3-5D induced transcription of the IFN-B promoter. IRF3-5D is a constitutively active mutant of IRF-3 [28] which activates IFN-B transcription in absence of additional stimuli. Silibinin but not SIL blocked IRF-3 activation of the IFN-B promoter (Figures 6C and 6D). Thus, the two mixtures differentially suppress inflammatory and antiviral signaling pathways.

### Anti-Inflammatory Effects of SIL and Silibinin on T Cells

**Figure 7:**
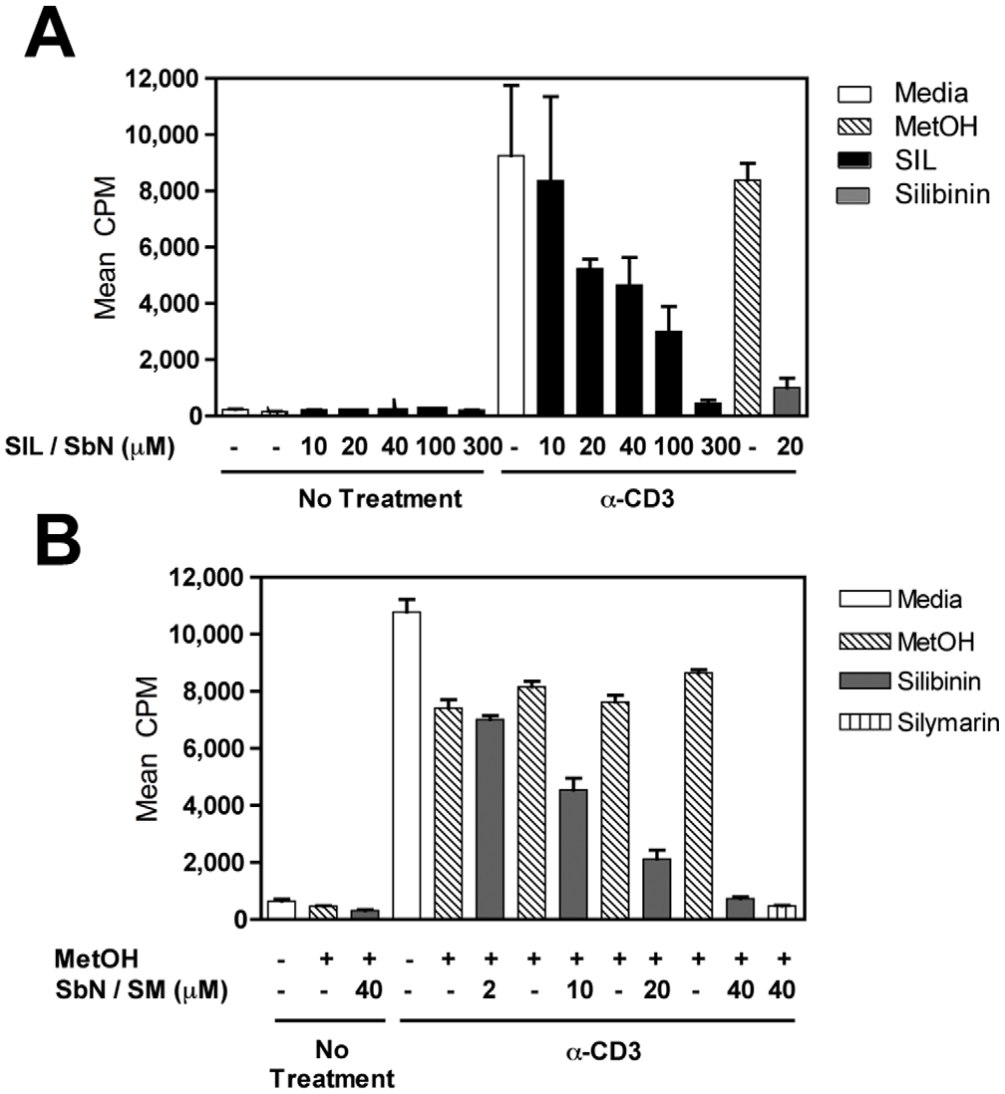
SIL and silibinin dose-dependently inhibit T cell proliferation. For all assays, a single dose of silibinin or SIL was added at culture initiation and compared to parallel cultures treated with a single dose of silibinin vehicle (MetOH) or media (control for SIL). Freshly isolated PBMC were tested for proliferative responses to plate-bound anti-CD3 (10 µg/mL) stimulation measured using ^3^H-thymidine incorporation. **A**, PBMC cultures treated with SIL at various doses (black bars) and the media control (white bar). Cells were also separately treated with silibinin control (gray bar), and the corresponding methanol control (hatched bar). **B**, PBMC cultures were treated with silibinin at various doses (gray bars), or with methanol solvent controls (hatched bars). Cells were also treated with silymarin as a positive control for inhibition of proliferation (bar with vertical lines). Open bars represent proliferation of PBMC stimulated with anti-CD3 alone in media, which serves as the control for SIL treatment. Results are shown as mean cpm incorporated. Error bars indicate 1 standard deviation among 3–4 replicates for each condition.

Given its lack of ability to inhibit NF-kB in hepatocyte cultures, we wondered whether SIL maintained the ability to inhibit PBMC proliferation, as shown previously for silymarin [12], [15]. SIL and silibinin dose-dependently inhibited PBMC proliferation induced by plate-bound anti-CD3 (Figure 7). The degree of inhibition was similar for both mixtures, with IC_50_'s of 12.3 µM and 12.9 µM for silibinin and SIL, respectively.

## Discussion

In the current study, we characterized the biological activities of oral (natural, ie silibinin) and intravenous (semi-synthetic, ie SIL) preparations from milk thistle on the HCV life cycle and inflammatory cellular functions that collectively contribute to development and progression of liver disease in hepatitis C. We demonstrate for the first time that SIL and silibinin elicit very different antiviral and anti-inflammatory actions in human liver and T cell cultures. Both mixtures inhibited virus fusion potently and NS5B RdRp activity weakly. SIL inhibited NS5B binding to RNA, whereas silibinin did not. Silibinin did not inhibit HCV RNA and protein expression from subgenomic genotype 1b or 2a replicons, but it did inhibit JFH-1 infection. In contrast, SIL inhibited 1b but not 2a subgenomic replicons and also inhibited JFH-1 infection at much higher doses than required for RdRp inhibition. Both silibinin and SIL inhibited progeny virus production from JFH-1 infected cells. Silibinin but not SIL inhibited innate inflammatory and antiviral signaling from NF-κB and IFN-B promoters. Both SIL and silibinin suppressed T cell proliferation to similar degrees. Overall, silibinin activity was similar to silymarin in all assays tested [13], [15], while SIL had a unique profile, requiring higher concentrations for antiviral activity in the JFH-1 system and was devoid of anti-inflammatory activity in hepatocyte cultures.

The effects of SIL and silibinin were tested against a widely used HCV genotype 1b isolate, BK, plus four patient-derived isolates with widely varying specific activities for RNA synthesis. Overall, both SIL and silibinin could inhibit the RdRps, with SIL being somewhat better than silibinin. However, inhibition plateaued at moderate drug concentrations, leading to maximal inhibition levels in most cases of only about 2–3 fold. The reasons for this unusual inhibition pattern are unknown. What is clear, however, is that SIL and silibinin do not shut down in vitro polymerase activity like nucleoside analog drugs do. Even at high concentrations of SIL or silibinin, the net RdRp activity is only marginally suppressed. We therefore posit that inhibition of NS5B polymerase elongation provides only a minor contribution to the log-fold suppression of viremia seen in patients receiving intravenous SIL. This conclusion is supported by the observation that in vitro HCV RdRp activity does not appear to be limiting for viral production in vivo, because no correlation was found between in vitro RNA polymerase activity and serum HCV titer in the patients from which the polymerases were derived [31].

Silibinin as well as Legalon-SIL are mixtures of two diastereoisomers, silybins A and B. These molecules are arranged around a flavonoid skeleton, and their overall structure is relatively hydrophobic. It is therefore possible that these molecules may act, at least in part, by incorporating or partitioning in the hydrophobic core of lipid membranes of both viruses and target (cell) membranes, as shown for similar compounds [32], [33]. This might lead to the stabilization of membranes by both molecules, which would in turn become less prone to fusion. This behavior would be reminiscent of that of arbidol, a broad-spectrum antiviral inhibiting HCV entry, membrane fusion and replication, as recently described by our group ([30], [34]; Teissier & Pécheur, unpublished observations).

SIL was as efficient as silymarin at inhibiting HCV-mediated fusion, with IC50 of ca. 6 µM and 5 µM respectively (see also [11]). Interestingly SIL displayed a much more potent inhibitory effect on HCV fusion than silibinin (IC50 6 µM vs ca. 25 µM, respectively). This indicates that silybins A and B by themselves are at least as efficient at blocking fusion as is silymarin, the mixture of multiple flavonolignans (including silibinin). However the chemical formulation of these molecules appears to alter pharmacological and antiviral activity, since SIL, the disuccinate form of silibinin, appears to be a more potent antiviral in the clinic than silibinin. Further studies are needed to determine if the chemical composition of SIL enhances its interaction with membranes.

HCV infection induces inflammation via hepatocellular sensing of virus by PRRs [35], [36], induction of oxidative stress [37], [38], [39], [40], [41], [42], and induction of inflammatory cytokines and chemokines [27]. Unfortunately, this response, which is usually beneficial to the host, is deregulated in chronic hepatitis C because the virus is not cleared. Inflammatory events such as T cell infiltration of the liver, and release of inflammatory cytokines and chemokines damage the liver via further induction of oxidative stress. Perpetuation of this inflammatory cascade and immune cell mediated liver damage is thought to induce subsequent fibrosis. Thus, chronic hepatitis C may be thought of as a disease caused by an inflammatory response gone awry [43], [44], [45], [46], [47]. In this harsh environment, hepatocytes die and regenerate more frequently. Since chronic inflammation is mechanistically involved in the establishment of cancer [48], and in particular hepatocellular carcinoma [49], the deregulated cellular responses are an integral part of the complex processes that lead to HCV-induced liver disease. We have shown that silymarin displays antioxidant, anti-inflammatory, antiviral, and immunomodulatory properties [12], [13], [50]. Therefore, we propose that silymarin elicits hepatoprotection by multiple actions that collectively reduce inflammation by several mechanisms including inhibition of NF-κB signaling, T cell proliferation and inflammatory cytokine production, and virus infection. Regarding anti-HCV effects, although silymarin and purified flavonolignans can inhibit NS5B polymerase activity, we propose that blockade of viral targets is not the dominant antiviral mechanism. Instead, silymarin blockade of cellular targets may confer antiviral effects by blocking virus entry, HCV RNA and protein expression, and virus transmission [11], [12], [15].

In summary, our data clearly demonstrate that silibinin and SIL function in different ways to induce hepatoprotection. SIL has shown antiviral efficacy during liver transplantation for end-stage hepatitis C [19] and for prior non-responders to pegylated IFN plus ribavirin therapy [18]. However, silymarin, silibinin, and SIL have been shown to modulate the expression and activity of various drug-metabolizing enzymes [51], [52], [53]. Given that much higher plasma and presumably liver levels of silymarin flavonolignans can now be achieved via oral [21] and intravenous [54] dosing, careful considerations should be given to the routes of administration, chemical composition, and possible interactions of pharmaceuticals with silymarin-derived flavonolignans [55]. Our studies suggest that future clinical and basic research studies of specific silymarin components, including those that are chemically modified, will be the key to understanding their clinical effects and developing novel and effective natural product-derived medicines for liver disease.
